# Convolutional Model with a Time Series Feature Based on RSSI Analysis with the Markov Transition Field for Enhancement of Location Recognition

**DOI:** 10.3390/s23073453

**Published:** 2023-03-25

**Authors:** Hyunji Lee, Jaeho Lee

**Affiliations:** 1 Department of IT Media Engineering, Duksung Women’s University, 33, Samyang-ro 144-gil, Dobong-gu, Seoul 01369, Republic of Korea; 2 Department of Software, Duksung Women’s University, 33, Samyang-ro 144-gil, Dobong-gu, Seoul 01369, Republic of Korea

**Keywords:** RSSI, indoor positioning, Markov transition field, CNN, time series data

## Abstract

Although numerous schemes, including learning-based approaches, have attempted to determine a solution for location recognition in indoor environments using RSSI, they suffer from the severe instability of RSSI. Compared with the solutions obtained by recurrent-approached neural networks, various state-of-the-art solutions have been obtained using the convolutional neural network (CNN) approach based on feature extraction considering indoor conditions. Complying with such a stream, this study presents the image transformation scheme for the reasonable outcomes in CNN, obtained from practical RSSI with artificial Gaussian noise injection. Additionally, it presents an appropriate learning model with consideration of the characteristics of time series data. For the evaluation, a testbed is constructed, the practical raw RSSI is applied after the learning process, and the performance is evaluated with results of about 46.2% enhancement compared to the method employing only CNN.

## 1. Introduction

Recognizing location indoors has attracted attention for well-positioning under the non-global positioning system (non-GPS) environment. Numerous studies have achieved unstable RSSI measurement because the use of RSSI does not require extra measuring methods such as a laser, camera, magnetic or acoustic sensor, or lidar. However, RSSI is not appropriate for recognizing location owing to its instability and unreliability [1,2,3,4,5,6]. Nonetheless, the powerful advantage of the method using only RSSI cannot be ignored because of its economic feasibility.

Although many studies have tried to find a solution for position estimation indoors, there is no representative method when using RSSI signals. Hence, we found an appropriate solution using neural networks. However, we found that traditional models such as recurrent or convolutional networks have limited performance due to the characteristics of unstable RSSI signals. Thus, we needed to make a method to find features from RSSI in an indoor environment to obtain some characteristics from stationary indoor objects.

Recently, only a few studies [7,8,9,10,11,12,13,14] have attempted to determine a solution using neural networks. Considering the characteristics of RSSI, i.e., time series data, several of them have approached the stream of a recurrent party, e.g., vanilla recurrent neural networks (RNNs) [15], long short-term memory (LSTM) [16], and gated recurrent unit (GRU) [17].

Although indoor environments can follow some characteristics, including stationary radio conditions and propagation status, components such as the walls, doors, furniture, equipment, and other structures generally do not move unless acted on by people. Therefore, some points of feature depend on indoor positions. Hence, focus must be placed on determining the solution by considering convolutional neural networks (CNNs), which are advantageous for finding and extracting a feature.

Methods utilizing CNN are appropriate and favorable for image processing. Numerous models employing the CNN approach focus on extracting visible pictures to discover features. As a perspective of convolution calculation in CNN, at least two or more dimensions are required to determine an effective feature. This leads numerous CNN-based models to image-relative areas with their significant performance. Furthermore, extracting effective features from time-dependent and serialized data using CNN is challenging because most of them have 1D characteristics with a constant sequence; RSSI is representative. A transition scheme of imagefication from time series data, i.e., converting RSSI to 2D data, is fundamental for utilizing CNN to find features from RSSI for indoor localization. Therefore, this study focused on the Markov transition field (MTF) [18] to transform images from serialized RSSI values.

Meanwhile, owing to the unpredictable fluctuation of RSSI, the effectiveness of the localization-based learning model using RSSI cannot guarantee performance compared with other areas such as image recognition. Additionally, the robustness of the output from the learning model must be enhanced, and location must be recognized for the RSSI domain. Therefore, an image transition scheme based on MTF with a noise injection technique must be investigated.

Several RSSI-based CNN models for recognizing indoor location [19,20,21,22], as discussed in the subsection of the next section, extract features from the radio map. However, they do not consider the characteristics of the time sequence phenomenon. Considering CNN, the transformation of imagefication may lose the time domain, except for in the spectrogram approach. Recurrent networks can be combined with CNN as a possible solution; however, this may cause complex computations. This study, to address the above-mentioned issues, proposed (*i*) a transformation scheme of imagefication based on MTF from RSSI with artificial injection of Gaussian noises and (*ii*) an appropriate CNN model considering the time series with MTF, excluding the recurrent perspective.

## 2. Related Work

To determine the appropriate features from the time series data using the CNN approach, one researcher investigated a method of image transformation from data, while others focused on the model structure. During image transformation, Ref. [23] calculated the magnitude of accelerations from each axis in three dimensions and then extracted a recurrent plot (RP) in the image domain from the time series data. Although the work was not aimed at recognizing location because the authors used RP with a simple CNN to determine motion class, a similar approach from the viewpoint of image transformation from serialized data was utilized. However, the fundamental issue with RP is classifying the threshold level, which is deterministically difficult and very subjective.

Another study detected the incident of a flat wheel caused by a railway breaking event [24]. The authors utilized Gramian angular field (GAF) [18] and proposed a single frequency-domain GAF (FDGAF) by transforming the vibration signals to a feature image in the frequency domain. They successfully obtained a meaningful feature on an image by addressing the dependency of frequency position; however, utilizing RSSI presents a limit. Although amplitude is significant in calculating distance through RSSI, it is not explicit for analysis in the frequency domain.

**Figure 1:**
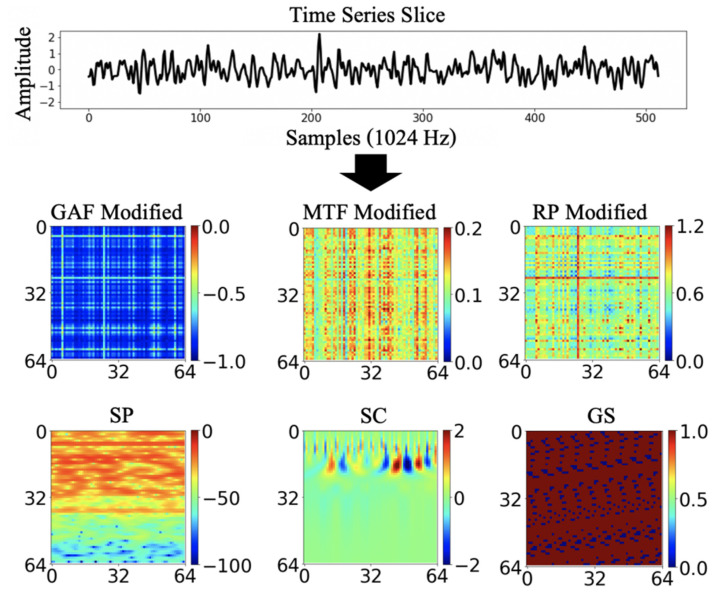
Comparison of relative transformation schemes from the same sample of RSSI data in [24]; results of GAF on the left, MTF in the center, and RP on the right.

Another study [25] investigated the condition of industrial assets using image transformation from temporal signals. They intensively analyzed several image encoding schemes consisting of spectrogram, scalogram, GAF, MTF, RP, and grayscale to evaluate the effectiveness of deep learning on image processing. Figure 1 shows examples of the transformation of time series data into the corresponding images by GAF, MTF, and RP. They concluded that most of the given algorithms exhibit reasonable performance; however, they could not conclude that one is a powerful method in various areas.

Another study performed model enhancement based on CNN with image transformation from an RSSI sequence. The authors of [19] developed an indoor positioning scheme using the fingerprint approach. They converted Wi-Fi signals and measured magnetic values on the corresponding images, then found features using the CNN model. Three-dimensional meanings could be found in the converted image: first, the length of measurement from the sensors; second, multiple features; and third, the equivalent process of the first stage. This was achieved with reasonable performance by combining heterogeneous signals. However, the absence of time dependency on the obtained feature is a limitation of the work.

The authors of [20] trained and extracted a feature using CNN from the RSSI data collected from the UJiindoorLoc [21] dataset. This focused on finding the dedicated building and floor level in large-scale sight as well as a relative indoor position. However, finding an appropriate feature presented a limit as the image for learning was constructed by only joining the pieces of RSSI, and the time series characteristics were not considered.

Finally, Ref. [22] suggested a CNN model to recognize a position in an indoor Wi-Fi environment with RSSI and performed a comparative study with some known models, namely, AlexNet [26], ResNet [27], ZFNet [28], Inception v3 [29], and MobileNet v2 [30]. Additionally, Ref. [31] enhanced performance in terms of computation power; although they accomplished robust performance by considering RSSI variations from Wi-Fi signals in a complex indoor environment, they missed time dependency from serialized data.

## 3. Image Transformation and Description of the Learning Model

### 3.1. Investigation of CNN Utilization with RSSI Variations

**Figure 2:**
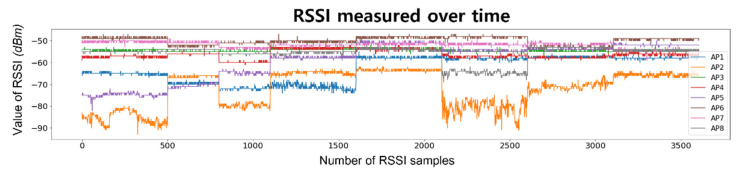
RSSI variation over time in the experimental environment.

**Figure 3:**
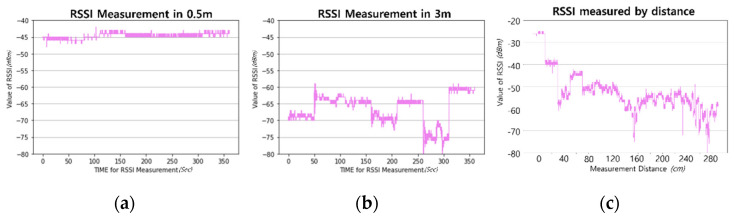
RSSI variations by practical measurement: from leftmost, (**a**) short distance, (**b**) long distance, and (**c**) various distances.

RSSI should be considered when constructing a location recognition system primarily because it can relatively and dependently decrease with increasing distance, that is, it is based on the hypothesis that the decrease in RSSI is inversely proportional to the square of the distance. The majority of researchers with experience contacting localization themes understand that utilizing RSSI is highly challenging. Considering Figure 2 and Figure 3c, nobody can ignore this issue.

Referring to [32], the RSSI value can be changed by various obstacles, as well as distance, temperature, humidity, and other interference on the same frequency. Figure 2 shows the RSSI variations collected in the experimental environment. Over a period of three months, RSSI was collected at intervals of approximately two weeks. At each measurement, about 75,000 RSSIs were collected from 150 cells. When the RSSI value is relatively high for one of the eight APs, indicating that RSSI is received from a nearby AP, the RSSI variation over time remains stable. However, when an RSSI is received from a relatively distant AP, the RSSI exhibits instability with rapid fluctuations.

Although the difference in the mean value of RSSIs between short and long distances is explicitly presented, as shown in Figure 3a,b, it is insufficient to estimate distance with the RSSI, as shown in Figure 3c. When RSSIs are measured over a long period of time and the meaningful values are averaged by removing the critical error, the estimated distance can be obtained. However, the system for location recognition should be operated in real time or provide a short response time. To meet this requirement, the abovementioned possibility, i.e., averaging long-time values, is impractical.

Nevertheless, RSSI can be significant for developing a positioning scheme from the viewpoint of a learning approach such as employing neural networks. Considering an indoor environment, numerous requisites, such as walls, furniture, equipment, facilities, and objects, are stationary.

The prima facie case from RSSI analysis can be considered an indicator derived from radio propagation, i.e., reflection, refraction, diffraction, scattering, and deep fading. Additionally, indoor environments can aggravate these indicators owing to the numerous obstacles and complex constructures. However, in the CNN domain, this condition can help find features because the indicators of radio propagation may be irregular but constant owing to the fixed obstacle. Therefore, herein, peculiar points from the RSSIs were assumed with high probability depending on the position. This case can make the CNN favorable.

**Figure 4:**
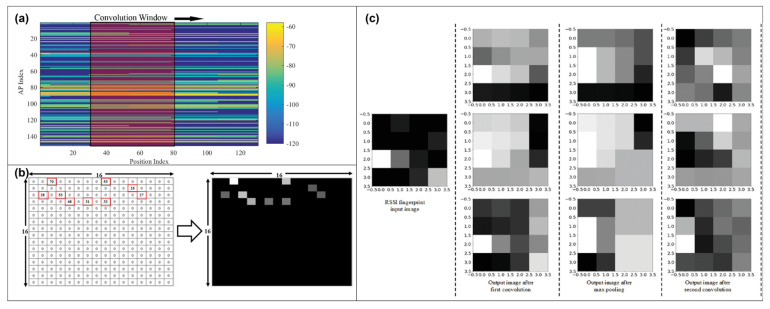
Examples of serialized RSSI transformation for CNN analysis on (**a**) [19], (**b**) [21], and (**c**) [31].

For utilizing CNN, a 2D data-structure-like image is required, as is common knowledge; however, several previous studies generated one via linking and serializing RSSI without considering new lines in the image, such as in [19], as expressed in (1). Moreover, several works, representatively described in Section 2, used the amplitude value, as shown in Figure 4. Therefore, they have a limited point to find a feature effectively.

(1) $$\left[\begin{array}{cccc} RSS_{1,1} & RSS_{1,2} & \cdots & RSS_{1,T}\\ RSS_{2,1} & RSS_{2,2} & \cdots & RSS_{2,T}\\ \vdots & \vdots & \ddots & \vdots \\ RSS_{N,1} & RSS_{N,1} & \cdots & RSS_{N,T} \end{array}\right]$$

The approach in (1) may easily lose the time dependency of RSSIs in the event of a new line in an image. Moreover, others, such as those in [19,21,31], may exhibit low effectiveness of training when the fluctuation of the RSSI variation becomes severe, and CNN may hardly probe time dependency in nature. Thus, an alternative solution is needed: a transition-based transformation method for image generation from RSSI, a solution reserving robustness against RSSI variations, and a model of CNN considering time series dependency.

### 3.2. Process for RSSI Imagefication

To employ CNN with RSSI, a transformation scheme for imagefication from 1D time series data to 2D image data is required. The RP, GAF, and MTF schemes are utilized to transform the 1D data into *m*–dimensional data.

RP transforms the time-dependent data into trace data in the space domain and then generates an image by calculating the distance between each point from the trace data located in the space domain. Additionally, RP compares the presented data with a threshold and marks 1 when the data are less than the threshold or 0 when the reverse takes place, as shown in (2). Thus, the algorithm can transform an appropriate image including time dependency only if the threshold is optimal.

(2) $$R\left(i,j\right)=\{\begin{array}{c} 1\;if\;\|\vec{x}\left(i\right)-\vec{x}\left(j\right)\|\;\leq \varepsilon \\ 0\;\;otherwise \end{array}$$

GAF transforms the serialized data to the value on the polar ordinate system by transforming the time stamp and time series data to a radius and cosine value, respectively, instead of the previous Cartesian coordinate system. The curve of the transformed value bends according to the increment of the time stamp. After transforming the image by summing or subtracting the angle on the polar ordinate system, the two schemes reserving the time dependency, the Gramian summation angular field and the Gramian difference angular field, are generated.

The results of [21] indicate that the results of the F1-score and true positive rate in MTF are predominant compared with RP and GAF. This suggests that MTF is suitable for finding a feature from the serialized RSSI, even if the results on them could depend on the characteristics of the test environment. Hence, in this study, the MTF algorithm was employed.

**Figure 5:**
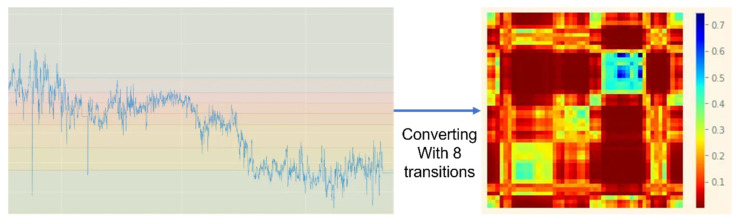
Example of transforming the serialized data to the corresponding image based on MTF with eight-level transitions.

MTF is one of the imagefication schemes. As shown in Figure 5, the overall range of amplitude is divided into some levels, indicating the transition numbers in MTF. The figure shows an example of an eight-level transition. The color map on the right side of the figure presents the amount of state transition events based on the levels; thus, MTF presents the transition probability field of each data point. It first divides 1D data, *x*, into the number of condition states, *Q*. Next, it constructs the weighted matrix with size *Q* × *Q* according to the time axis. The regularization is performed by ensuring that the sum of each row in the matrix is set to 1. The Markov transition matrix, *W*, can be generated as shown in (3). The matrix is presented as the frequency of the *q_j_*{1 ≤ *j* ≤ *Q*} with the period of time series data, *x_i_*, at time stamp *t_i_*.

(3) $$W=\left[\begin{array}{ccc} w_{11}|P\left(x_{t}\in q_{1}|x_{t-1}\in q_{1}\right) & \cdots & w_{1Q}|P\left(x_{t}\in q_{1}|x_{t-1}\in q_{Q}\right)\\ \begin{array}{c} w_{21}|P\left(x_{t}\in q_{2}|x_{t-1}\in q_{1}\right)\\ \vdots \end{array} & \begin{array}{c} \cdots \\ \ddots \end{array} & \begin{array}{c} w_{2Q}|P\left(x_{t}\in q_{2}|x_{t-1}\in q_{Q}\right)\\ \vdots \end{array}\\ w_{Q1}|P\left(x_{t}\in q_{Q}|x_{t-1}\in q_{1}\right) & \cdots & w_{QQ}|P\left(x_{t}\in q_{Q}|x_{t-1}\in q_{Q}\right) \end{array}\right]$$

Evidently, from Equation (3), *W* loses the distribution of *x* and time dependency. To preserve the information on the time domain, it can be transformed into MTF by arranging the probabilities according to the sequence on the time domain. Equation (4) presents the Markov transition field, *M*. The value of the *i*^th^ row and *j*^th^ column, *M_ij_*, shows the probability that the state in the level, *q_i_*, at time stamp *t_i_* is transited to the state in *q_j_* at *t_j_*.

(4) $$M=\left[\begin{array}{ccc} M_{11} & \begin{array}{cc} M_{12} & \cdots \end{array} & M_{1n}\\ \begin{array}{c} M_{21}\\ \vdots \end{array} & \begin{array}{c} \begin{array}{cc} M_{22} & \cdots \end{array}\\ \begin{array}{cc} \;\;\;\vdots & \;\;\;\ddots \end{array} \end{array} & \begin{array}{c} M_{2n}\\ \vdots \end{array}\\ M_{n1} & \begin{array}{cc} M_{n2} & \cdots \end{array} & M_{nn} \end{array}\right]=\left[\begin{array}{ccc} w_{ij}|x_{1}\in q_{i},\;x_{1}\in q_{j}\; & \cdots & w_{ij}|x_{1}\in q_{i},\;x_{n}\in q_{j}\\ \begin{array}{c} w_{ij}|x_{2}\in q_{i},\;x_{1}\in q_{j}\\ \vdots \end{array} & \begin{array}{c} \cdots \\ \ddots \end{array} & \begin{array}{c} w_{ij}|x_{2}\in q_{i},\;x_{n}\in q_{j}\\ \vdots \end{array}\\ w_{ij}|x_{n}\in q_{i},\;x_{1}\in q_{j} & \cdots & w_{ij}|x_{n}\in q_{i},\;x_{n}\in q_{j} \end{array}\right]$$

For the RSSI transformation, the RSSI values were obtained during a window period and slid in a forward direction. Subsequently, each array was obtained from the window at each time, and then images were generated by transforming the arrays based on MTF. If we have *k* number of access points (APs) for the RSSI measurement, *x* can be presented as a vector matrix with time stamp *j* according to (5).

(5) $$x=\left[RSS_{1}^{1}\;RSS_{1}^{2}\;\cdots \;RSS_{1}^{k}\;RSS_{2}^{1}\;RSS_{2}^{2}\;\cdots \;RSS_{2}^{k}\;\cdots \;RSS_{j}^{1}\;RSS_{j}^{2}\;RSS_{j}^{k}\;\right]$$

### 3.3. Artificial Noise Injection into RSSI before Image Transformation

The value of RSSI can have different patterns depending on the complex indoor environment, including structural space, obstacle position, wall material, and humidity. For more robustness in the training effectiveness, artificial noise injection can enhance the performance of the learning model. Thus, Gaussian noise was injected into the RSSI signal before transforming it into an image.

The distance derived from RSSI can be generally presented as shown in (6), where *c* is a constant value that indicates the measured RSSI at a predefined criterion distance.

(6) $$R_{raw}=-10n\log_{10}d+c$$

Combining the artificial Gaussian noise with preprocessed RSSI values can yield a stable and robust estimation of distance when an unexpected change in radio propagation is incurred by training the network model with artificial fluctuations. According to the Gaussian distribution thesis, the probability density function can be a constant; thus, the distance can be denoted as shown in (7).

(7) $$R_{pro}=-10n\log_{10}d+\frac{1}{\sigma \sqrt{2\pi }}exp\left(-\frac{{\left(x-\mu \right)}^{2}}{2\sigma^{2}}\right)+c$$

Next, this is normalized by *MinMaxScaling* into both the original and noise-injected signals to avoid an increment in *R_pro_*. Finally, the composed RSSI with the artificial noise can be used as input data for the image transformation by MTF.

### 3.4. Design of the Training Model

**Figure 6:**
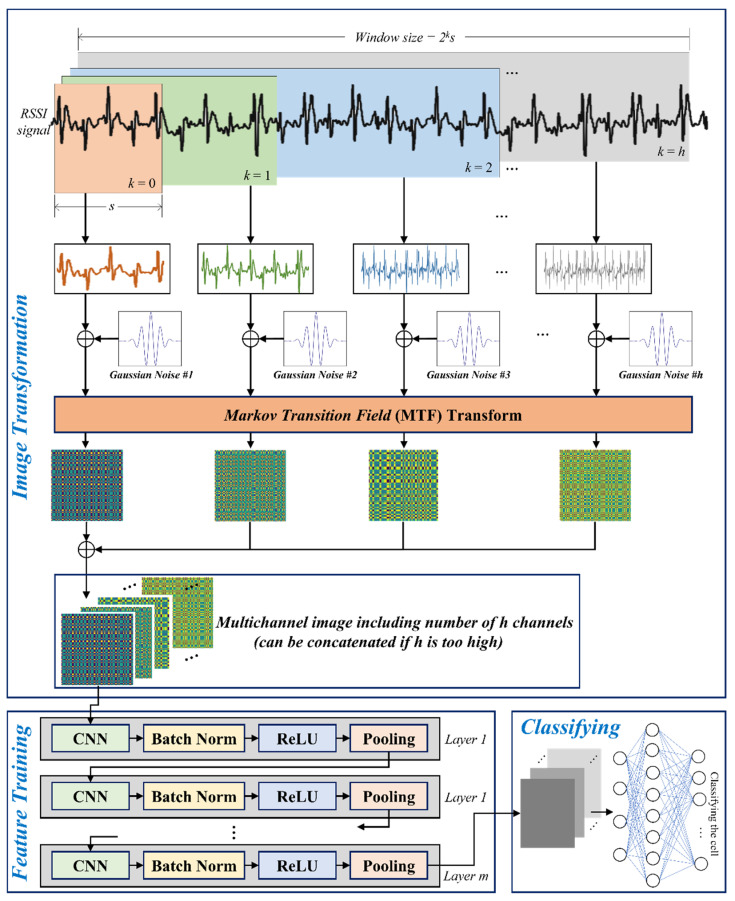
Process of training an image to decide indoor position through artificial Gaussian noise injection and MTF-based image transformation by concatenating multiple sliding windows.

The main goal of the proposed model to determine location is to maintain and consider the characteristics of time series sequences from RSSIs. Therefore, the CNN approach with multiple sliding windows was employed. As shown in Figure 6, the measured RSSI values are collected during each window classified into *k* types of sizes, where the value of each window (*s*, 2*s*, 4*s*) indicates the slots of {2^0^, 2^1^, 2^2^, … 2*^h^*} multiplied by the unit of time slot, *s*. At the next time stamp, all windows slide by one step while retaining their size. The windows are classified to maintain the characteristics of time dependency with a multiple range of time domains.

After the set of values in each window is modified with artificial Gaussian noise injection, the values at each window are transformed with MTF into images. That is, the number of images generated is seven if *h* is set to 2, i.e., four images on *k* = 0, two images on *k* = 1, and one image on *k* = 2, where *k* is the counter that can be maximized at *h*. This is the overall preprocess before learning the model. Notably, the image size from all preprocesses is equivalent considering the MTF mechanism.

**Figure 7:**
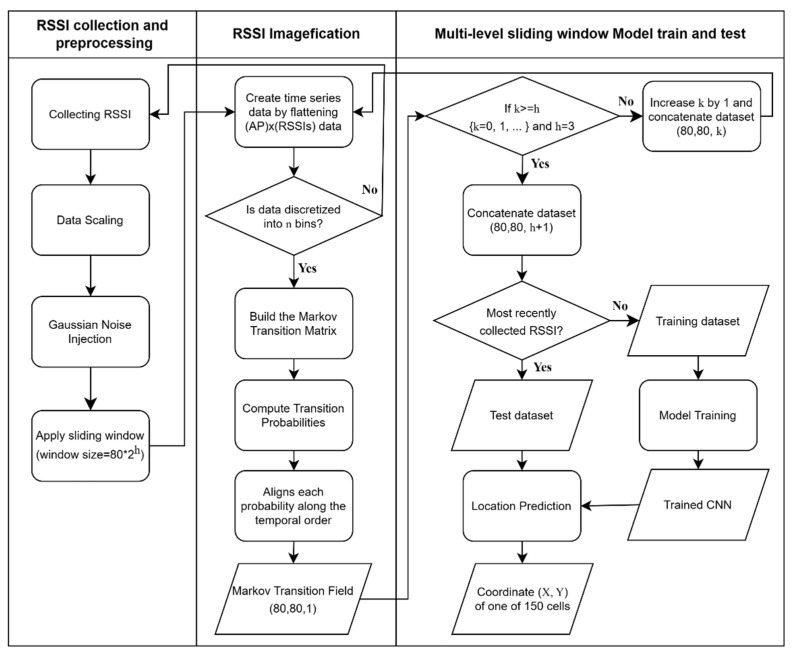
The overall process from the collection of RSSIs to the model training and testing.

In the model, the structure of a layer on the CNN part is composed of convolution, batch normalization, ReLU, and pooling sublayers. Further, *l* number of sublayers exist in the CNN part. In the initial step, the image data with multiple channels, *h*, derived from multiple windows, is input into the first convolution sublayer in layer 1. Additionally, some images are concatenated if *h* is set too high. The polling sublayer in each layer is connected to the convolution sublayer in the next layer up to layer *l*. Finally, fully connected networks (FCNs) with multiple-layer perceptron are applied as the classifier to determine the cell for the location indoors. Figure 7 presents the overall process, including RSSIs collection, preprocessing, artificial noise injection, MTF-based image transformation, training, and testing the model, as described above.

## 4. Evaluation

### 4.1. Testbed Environment and Dataset Construction

**Figure 8:**
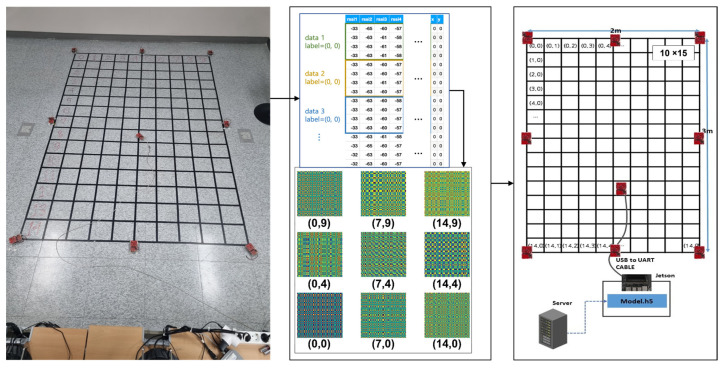
Environment of testbed for the experiment of indoor location recognition using the proposed approach.

To evaluate our approach in a practical scenario, a testbed environment was conducted, as shown in Figure 8. The testbed was composed of 150 cells (10 cells in the *x*-axis and 15 cells in the *y*-axis), and the unit of a square cell had a size of 20 cm in each axis. Eight APs, which acted as anchor points for the relative indoor position, were employed. A tag was positioned at the center of each cell and fixed in place to face the 12-o’clock direction, as shown in the left picture of Figure 6. By moving a tag, the practical RSSI was measured via a tag connected to the evaluation board, the Jetson Nano of nVidia, from the eight APs with a time interval of 50 msec per AP. The learning process was implemented on a GPU server, and then the model file was injected into the evaluation board for the experiment after the learning process.

**Figure 9:**
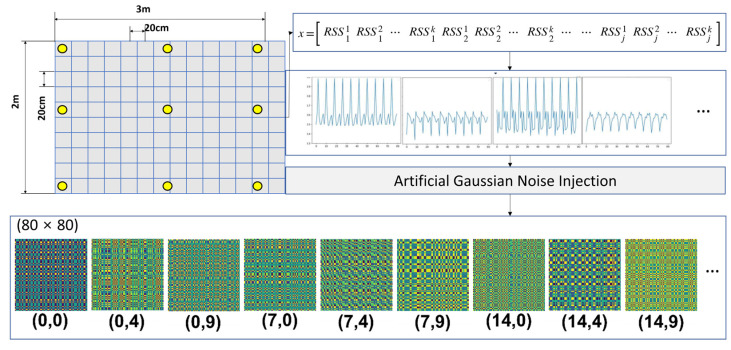
Examples of generated MTF images from the measured RSSIs from our testbed.

For the learning process, we used a GPU server including two nVidia Quadro RTX 8000 with 48 GB of graphic memory, an Intel Xeon W-4215 with 8 cores, 384 GB of system memory, and sufficient storage. Consequently, at the training and evaluation stages, the features were extracted, using the server by measuring RSSIs from the testbed. At the test stage, the results were obtained, using the evaluation board with the real-time RSSIs by moving the tag after injecting the model file, i.e., the *.h5 file generated by the GPU server, into the evaluation board, i.e., the nVidia Jetson Nano. It is worth noting that the final estimation process on-site was only operated on the evaluation board. Through this process, the transformed images were generated for every cell over time. Figure 9 shows some examples of images generated by MTF.

The training model was designed with configurations, including the hyperparameters summarized in Table 1, that can be classified into three perspectives: dataset, preprocessing, and learning. The first perspective describes the dataset configuration. All the datasets presented in the upper column are related to gathering of RSSIs via the testbed. They were measured for 6 days in an equivalent environment. Then, we separated the test set from the dataset, so the final test set used for performance evaluation was never used in the training step, although the testbed place was commonly used in the overall process. The configuration of the preprocessing perspective, presented in the middle column, indicates the overall process before the learning phase, i.e., the parameters for the following processes: RSSI measurement, sampling with sliding multiple windows, noise injection, and MTF transformation. The interval for image generation is defined based on parameters such as the RSSIs for an image and the RSSI interval; for instance, an interval of 2 s can be obtained when *k* and *s* are set to 2 and 1, respectively. It is worth noting that eight RSSIs are measured every 50 ms, as shown in Table 1.

The configuration of the learning perspective, presented in the lower row, indicates the overall process of feature extraction and classification, i.e., the parameters for the CNN model to find the features from the MTF-transformed images and for the classification model to determine an appropriate location cell in the testbed. Notably, both the number of images generated by MTF transformation and the number of channels inserted into the neural network parameters are equivalent to the value of *h*. They are presented in the preprocessing perspective in the table.

### 4.2. Analysis of the Testbed Results

To analyze the performance of the proposed scheme, the proposed model was implemented and tested with MTF transformation and artificial noise injection using the testbed. The fundamentally required test results are the accuracy of the location cell decision at each cell on the testbed. Based on Table 1, we previously gathered practical training data from the testbed, trained and validated them using our model, and then obtained the accuracy with 230 test sets of RSSI data that were excluded during the training process. It is worth noting that one set of RSSI data is composed of eight RSSIs from eight APs.

**Figure 10:**
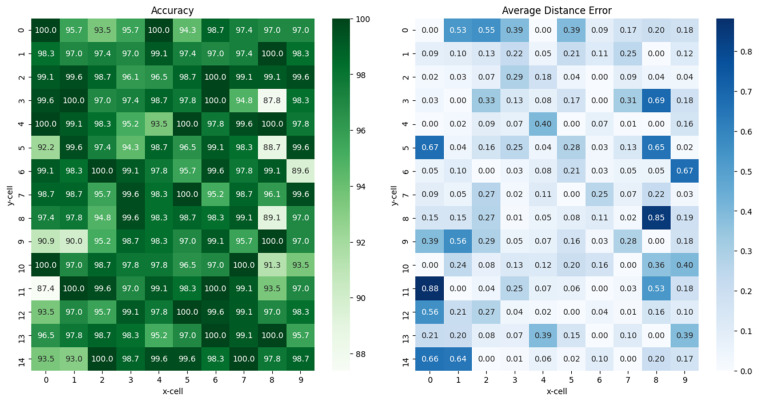
Results of the accuracies (**left**) and the distance errors (**right**) of position estimation by our learning model in each location cell via the constructed testbed.

The numbers shown in Figure 10 present the results of the averaged values: accuracies for each position decision on the left subfigure and distance errors on the right subfigure. The unit of average distance errors on the right subfigure are equivalent to the units of cell size. For instance, if the number is 2 in a given cell, the distance error between the actual position and the estimated position is twice the cell size, that is, a number 2 is equivalent to 40 cm if a cell has a width and height of 20 cm in the testbed. The distance error was obtained by averaging the Euclidean distances between the result and the label at each of the 230 test sets of RSSI data. The maximum accuracy was up to 100% for 21 out of 150 cells, and the minimum was 87.4% at (11, 0). It is important to note that the cell positions are denoted as (*y*, *x*).

**Figure 11:**
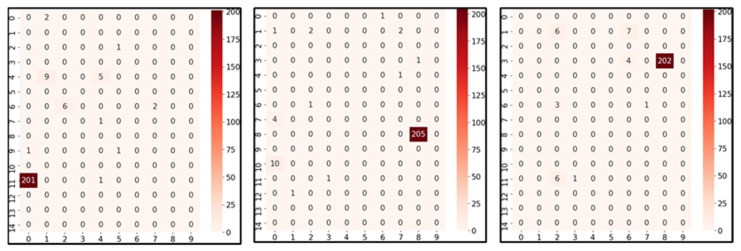
Results of the three worst positions among the 150 cells with highest distance errors in cells (11, 0) on the left, (8, 8) in the middle, and (3, 8) on the right of the figure.

The results shown in Figure 11 present the number of estimated positions for the worst three cells with the longest distance error, in (11, 0), (8, 8), and (3, 8), among the 150 cells. The actual position is located in a red box. The total number of test sets was 230. Each value in a cell presents the number of times the model estimated that position. For example, 201 in (11, 0) means that the model estimated the position in (11, 0) 201 times out of 230 test times, where the actual position was (11, 0).

Evidently, from the cells, most of the estimated results point out the actual cells, but some results have long-distance errors. During the prediction, the model decided the position depending only on features generated by MTF transformation, and the raw RSSI data were found to be highly unstable. Meanwhile, several results estimated the exact position, even considering the worst three cases. This can be overcome by determining a solution that considers the information about neighboring cells. Therefore, our next work aims to address this issue. These 3 are exceptional cases, while the other 147 cells have higher accuracies and lower distance errors than the aforementioned 3 cells.

**Figure 12:**
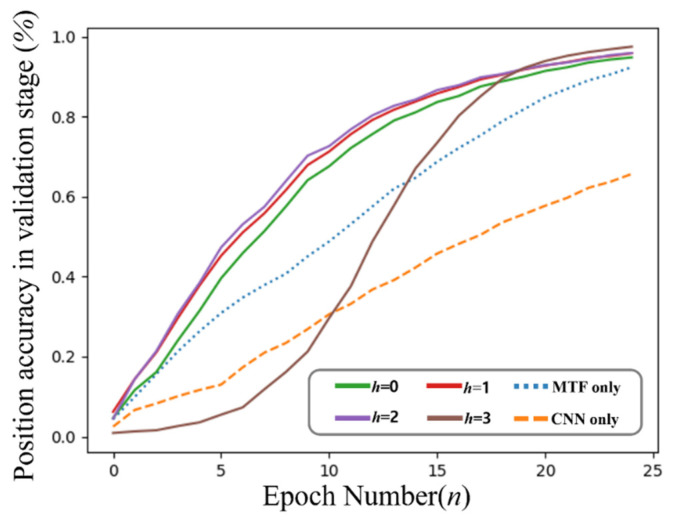
Results of the evaluation accuracy of position estimation 0 based on the increment of epochs with different standard deviations applied to the injected artificial noise, from the perspective of the *x*-axis (left) and *y*-axis (right) on the testbed.

The results shown in Figure 12 present the averaged evaluation accuracies of the position decisions based on the increment of the epoch number with the variable values of *h* presented in Section 3.4 and Figure 6 through the testbed. The results were obtained with varying *h* values; however, the other test conditions were maintained equivalently as presented in Table 1. All results presented with *h* employ both MTF and artificial noise injection, except for two lines. The results named “MTF only” exclude noise injection, and the results named “CNN only” employ neither MTF nor noise injection.

The lowest result is presented when evaluating “CNN only.” That is, “CNN only” uses raw RSSI converted to a simple 2D input shape to proceed with CNN learning. It approaches approximately 65.7%. In comparison, our main scheme approaches at least 94.85% with *h* = 0 and at most 97.54% with *h* = 3. “MTF only” approaches 92.4%. Evidently, the effectiveness of both MTF and noise injection are explicitly advantageous.

Table 2 summarizes the test results of accuracies and averaged distance errors of the proposed scheme, comparing two approaches: “MTF only” includes MTF transformation but excludes artificial noise injection, and “CNN only” employs the previous image transformation with serialized RSSIs as in (1) instead of MTF and noise injection. The accuracy results were obtained through binary classification by deciding the exact position for the explicit viewpoint. Meanwhile, the distance error results reflect the distance between the actual position and the estimated position. Therefore, when considering the number of the decision to select a neighboring cell, the results of the distance errors must be considered.

The obtained results revealed that our approach exhibited better performance and less distance error compared with the other two approaches. The effect of the artificial noise injection was remarkable, and the effect of MTF was distinctly presented. Observing the employment of MTF, although the difference in accuracy was not extremely high, the difference in distance error was explicitly present.

**Figure 13:**
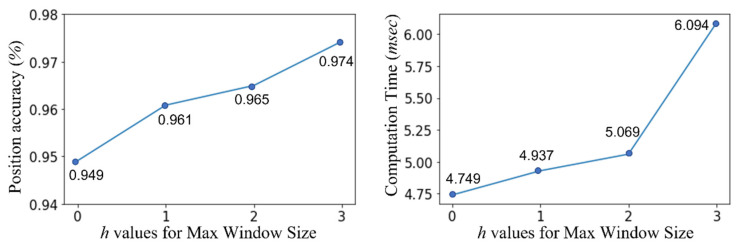
Results of average accuracies for position recognition and spent computation time according to the increment of maximum window size, *h*, through the testbed.

As presented in Section 3.4, the performance of the proposed scheme can induce different performance levels depending on the maximum window size derived from configuring the value of *h*. Hence, we conducted another experiment with the model on the testbed to obtain the results according to the increment of *h*. The results are presented in Figure 13. Additionally, higher *h* values can lead to higher performance owing to the large amount of information to be trained; however, this can aggravate the response time caused by high computation.

The left subfigure in Figure 13 shows the accuracy of the proposed scheme, and the right subfigure shows the spent time for the computation. Each result in the right subfigure presents the single computation time for a single test data under the evaluation board described in Section 4.1. Evidently, the value of *h* increased the position accuracy; however, the amount of gradient decreased gradually. Conversely, the results of the computation time required for a given value of *h* exhibited a growing slope, but the gradient increased gradually. Synthetically, an appropriately optimized point must be determined. Evidently, setting a size of 2 can be considered optimal in the given configuration. Notably, the optimal value is not absolute and can be redefined depending on the H/W environment or parameter configurations.

**Figure 14:**
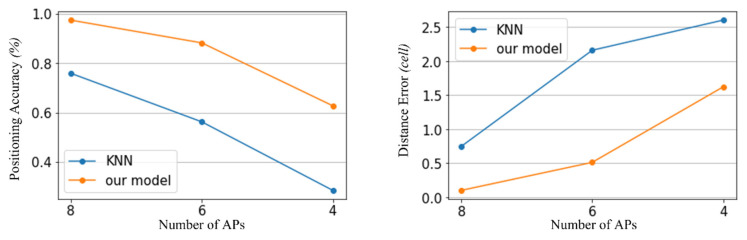
Comparison results between the proposed scheme and the algorithm-based fingerprinting method (KNN).

Additionally, we conducted another experiment to compare the proposed scheme to the traditional fingerprint algorithm, varying the number of APs. For the traditional fingerprint algorithms to estimate the location recognition, *k*-Nearest Neighbor (KNN) and cosine similarity have been widely considered the representative methods. We employed KNN for the comparison considering that it has been widely utilized in the location-based system. In Figure 14, two schemes present that the performance decreases as the number of APs decreases. However, considering the overall number of APs, the proposed scheme shows better performance in terms of position accuracy and distance error.

## 5. Conclusions

In this study, to enhance location recognition in indoor environments, a learning scheme including MTF image transformation from serialized RSSI data and artificial Gaussian noise injection was presented. The scheme considers image transformation to find appropriate features from RSSIs while maintaining the characteristics of serialized time series data; therefore, it can estimate the corresponding position using the CNN approach. The value of the scheme is also demonstrated by the results of its performance after its implementation on the constructed testbed. To provide a more comprehensive evaluation, we will continue the experiments by expanding our testbed to include large environments with non-LOS conditions. Further studies are aimed to find a solution for the method of artificial noise generation, including the use of various distributions, controlling the variance for the noise with multilevel RSSIs, and utilizing an auto-encoder. Additionally, other approaches are also planned for our future works, such as regression methods and localization for crowded agents.

## Figures and Tables

**Table 1:** Description of the hyperparameters for training our model.

Class	Parameters	Configurations	Parameters	Configurations
Dataset	Total RSSIs	465,000	RSSI interval	50 ms/AP
	RSSIs per cell	3100	No. of APs	8
	Train:Val:Test	7:1:2	No. of cells	150
Preprocessing	Gaussian Noise	*N* (0, 0.5)	RSSIs for an image	80 × 2^k^s
	*h* for Max Windows	3	Sliding step	2 samples
	Length/Window	80	Input size	(80, 80, 4)
	No. of levels in MTF	3	Transformation time	≤1 ms/pic
	Generated Images	1150/cell	Measured RSSI data	4000/cell
Learning	Learning rate	0.001	Batch size	32
	Optimizer	Adam	Epoch	25
	Loss function	Crossentropy ^1^	No. of layers	10
	Classifier	FCN	No. of classes	150
	Normalizer	Batch norm	Early stop	Yes

^1^ sparse_categorical crossentropy.

**Table 2:** Results of position accuracy and distance error on our model for the “MTF only” and “CNN only” methods.

Result Type	MTF + Noise Injection	MTF Only	CNN Only
Accuracy (%)	97.43	92.29	66.63
Distance Error (cell)	0.1	0.33	1.1

## Data Availability

Not applicable.
